# The Clinical Relevance of Hypothyroidism in Patients with Solid Non-Thyroid Cancer: A Tantalizing Conundrum

**DOI:** 10.3390/jcm11123417

**Published:** 2022-06-14

**Authors:** Maria V. Deligiorgi, Dimitrios T. Trafalis

**Affiliations:** Department of Pharmacology—Clinical Pharmacology Unit, Faculty of Medicine, National and Kapodistrian University of Athens, 75 Mikras Asias Str., Goudi, 11527 Athens, Greece; dtrafal@med.uoa.gr

**Keywords:** cancer prognosis, cancer risk, hypothyroidism, levothyroxine, liothyronine, solid non-thyroid cancer, thyroid hormones, thyroid hormone receptors, thyroid stimulating hormone

## Abstract

Hypothyroidism in patients with solid non-thyroid cancer is a tantalizing entity, integrating an intriguing thyroid hormones (THs)–cancer association with the complexity of hypothyroidism itself. The present narrative review provides a comprehensive overview of the clinical relevance of hypothyroidism in solid non-thyroid cancer. Hypothyroidism in patients with solid non-thyroid cancer is reminiscent of hypothyroidism in the general population, yet also poses distinct challenges due to the dual role of THs in cancer: promoting versus inhibitory. Close collaboration between oncologists and endocrinologists will enable the prompt and personalized diagnosis and treatment of hypothyroidism in patients with solid non-thyroid cancer. Clinical data indicate that hypothyroidism is a predictor of a decreased or increased risk of solid non-thyroid cancer and is a prognostic factor of favorable or unfavorable prognosis in solid non-thyroid cancer. However, the impact of hypothyroidism with respect to the risk and/or prognosis of solid non-thyroid cancer is not a consistent finding. To harness hypothyroidism, or THs replacement, as a personalized anticancer strategy for solid non-thyroid cancer, four prerequisites need to be fulfilled, namely: (i) deciphering the dual THs actions in cancer; (ii) identifying interventions in THs status and developing agents that block tumor-promoting THs actions and/or mimic anticancer THs actions; (iii) appropriate patient selection; and (iv) counteracting current methodological limitations.

## 1. Introduction

The integration of the endocrine perspective into the current paradigm shift in cancer care toward a multidisciplinary initiative [1] has nurtured interest in the clinical relevance of hypothyroidism in the setting of cancer.

Hypothyroidism is one of the most common endocrine disorders [2,3,4], defined as a deficiency of thyroid hormones (THs), especially of 3,3′,5,5′-tetraiodo-L-thyronine (T4) and 3,5,3′-triiodo-l-thyronine (T3).

Despite its long history, spanning over two millennia [5,6,7], hypothyroidism remains an interesting field of research due to the pivotal role of THs in every physiological process and in many pathological conditions, including cancer. Originally conceived in 1896 [7], the THs–cancer association was only sporadically explored until 1986, when the cloning of the complimentary DNA(s) (cDNA(s)) that encode nuclear THs receptor(s) (TR(s)) [8] informed the understanding of the classical actions of THs as transcription factors. Subsequently, the identification of TRs at the transmembrane protein integrin ανβ3 [9] in 2005 paved the way for the recognition of nonclassical THs actions mediated by diverse extranuclear TRs. To date, major advances in research have consolidated the dual role of THs in cancer—tumor-promoting versus anticancer—exerted through the coordination of classical and nonclassical THs actions [8,9,10,11,12,13,14,15]. The landscape of THs actions is further complicated by the emerging role of THs metabolites in cancer [16,17]. The clinical relevance of hypothyroidism in patients with solid non-thyroid cancer shares many complex characteristics with hypothyroidism in the general population [18,19], but the former is even more complicated than the latter due to distinct issues arising from the hypothyroidism–cancer association [20,21,22,23,24,25,26,27,28,29,30,31,32,33,34].

A notable number of cancer patients are expected to confront the hypothyroidism–cancer association, given that hypothyroidism constitutes a common adverse effect of innovative anticancer treatments [20,21,22,23,24,25,26,27,28,29,30,31,32,33] and diagnostic procedures using iodinated contrast media [34], which have, currently, revolutionized the standard of cancer care. Hopefully, hypothyroidism that is induced by the administration of iodinated contrast media is not general and is transient, being attributed to the suppression of THs synthesis via the Wolff–Chaikoff mechanism—an effect that lasts only a few weeks. On the contrary, hypothyroidism—primary or secondary—related to innovative anticancer treatments can often be permanent.

With an increase in the survival rates of most types of solid non-thyroid cancer, clinicians are urged to prioritize the maintenance of patient quality of life, which is subverted by hypothyroidism. The prompt personalization of the diagnosis and treatment of hypothyroidism in patients with solid non-thyroid cancer necessitates close collaboration between oncologists and endocrinologists. Of paramount clinical importance, hypothyroidism in patients with solid non-thyroid cancer has been indicated to be a predictive factor with respect to decreased or increased cancer risk and as prognostic factor of favorable or unfavorable cancer prognosis. However, the impact of hypothyroidism on the risk and/or the prognosis of solid non-thyroid cancer is not a consistent finding. Such inconclusive data hamper, so far, the exploitation of hypothyroidism as an anticancer strategy despite initial enthusiasm about the anticancer efficacy of myxedema coma in metastatic non-small-cell lung cancer (NSCLC), arising from a hallmark case report in 1993 [35].

The present review provides an overview of the clinical relevance of hypothyroidism in patients with solid non-thyroid cancer. Background knowledge on the molecular aspects of THs actions is briefly discussed as the rationale for the clinical relevance of hypothyroidism in cancer. Major issues related to the diagnosis and treatment of hypothyroidism in patients with solid non-thyroid cancer are critically discussed. The present review recapitulates the latest clinical studies addressing the predictive and the prognostic value of hypothyroidism in the setting of solid non-thyroid cancer. Finally, it highlights current challenges and future perspectives with regard to harnessing hypothyroidism, or THs replacement, as an anticancer strategy for patients with solid non-thyroid cancer.

## 2. Methods

The key questions of the search query were: (1) “What is the molecular background of THs actions in cancer?”; (2) “How is hypothyroidism in patients with solid non-thyroid cancer diagnosed and treated?”; (3) “How is hypothyroidism in patients with solid non-thyroid cancer related to cancer risk and cancer prognosis?”; and (4) “How can hypothyroidism in patients with solid non-thyroid cancer be exploited in cancer therapeutics?” The search of literature was conducted using the following electronic databases: PubMed, Google Scholar, Scopus.com (www.scopus.com), ClinicalTrials.gov (www.clinicaltrials.gov), and the European Union Clinical Trials Register from January 2011 to November 2021. Certain historical articles related to this issue published before 2011 were searched and retrieved. Search terms referred to “THs actions in cancer”, “hypothyroidism in cancer patients”, “solid non-thyroid cancer”, and “anticancer treatment-induced hypothyroidism”. The following types of articles were included in our search: case series reports, research articles, narrative and systematic reviews, meta-analyses, expert committee guidelines, and editorials. To narrow the search, we used the following search blocks: THs actions AND cancer hallmarks, anticancer treatment AND hypothyroidism, solid non-thyroid cancer risk AND hypothyroidism, solid non-thyroid cancer prognosis AND hypothyroidism. Full articles from the selected literature were retrieved and assessed thoroughly. The purpose of our review is to synthesize the literature concerning hypothyroidism in patients with solid non-thyroid tumors and highlight the challenges that need to be overcome through future research.

## 3. Molecular Aspects of the Implication of THs in Cancer as the Rationale for the Clinical Relevance of Hypothyroidism in Cancer Patients

To understand the clinical relevance of hypothyroidism in cancer, it is essential to delve into the role of THs in cancer, which is integrated via diverse THs actions. Historically, THs actions have been classified as “genomic” (classical), mediated through nuclear TRs, and “non-genomic” (nonclassical), mediated through extranuclear TRs located in the cytoplasm, at the transmembrane protein integrin αvβ3, or at other sites of the plasma membrane. Nowadays, it has been acknowledged that “non-genomic” THs actions can affect transcription. Therefore, a novel classification of THs actions in four types based on the involved molecular pathways is gaining ground [36].

Type 1 comprises the TR-dependent signaling of THs mediated by the direct binding of T3-liganded nuclear TR to DNA. Two different genes located on two different chromosomes encode two TR types (TRα, TRβ), while alternative splicing gives rise to several TR isoforms, which are widely distributed in a tissue-specific manner and form heterodimers with retinoid X receptor (RXR). The latter regulate the expression of target genes through binding to specific DNA sequences located in THs response elements (TRE). TR/RXR heterodimers are nonpermissive, forming complexes with corepressors of transcription. The binding of T3 to TRs leads to the dissociation of corepressors from the TR/RXR heterodimers and the recruitment of coactivators, inducing the transcription of various target genes implicated in development, differentiation, and metabolism in a tissue/cell-specific manner. TRs can also suppress gene expression. T3 shows a higher binding affinity for TR compared to T4 [13,36,37].

Type 2 comprises the TR-dependent signaling of THs mediated by the indirect binding of T3-liganded nuclear TR to DNA through tethering to other chromatin proteins that act as transcription factors [36].

Type 3 comprises the THs-signaling initiated by the interaction of T3 with cytoplasmic TR, or with TR located at the plasma membrane. 

TRβ1 rapidly activates the phosphoinositide 3-kinases (PI3K) pathway, leading to transcription of downstream target genes (e.g., hypoxia-inducible factor 1-alpha (HIF-1α), Glucose transporter 1 (GLUT1), platelet isoform of phosphofructokinase (PFKP) genes). The interaction of T3 with the truncated isoform p30 TRα1 at the plasma membrane inactivates the transcription of target genes via the binding of PI3K to p85α and activates various transduction proteins (e.g., type II cyclic guanosine monophosphate (cGMP)-dependent protein kinase (PKGII), extracellular signal-regulated kinases (ERK)), and the nitric oxide synthase (NOS). T3-liganded TRβ1 at the plasma membrane initiates the activation of the cytoplasmic mitogen-activated protein kinase (MAPK)/ERK1/2 cascade, which in turn activates the sodium proton exchanger (Na^+^/H^+^) at the plasma membrane [12,13,15,36].

**Figure 1:**
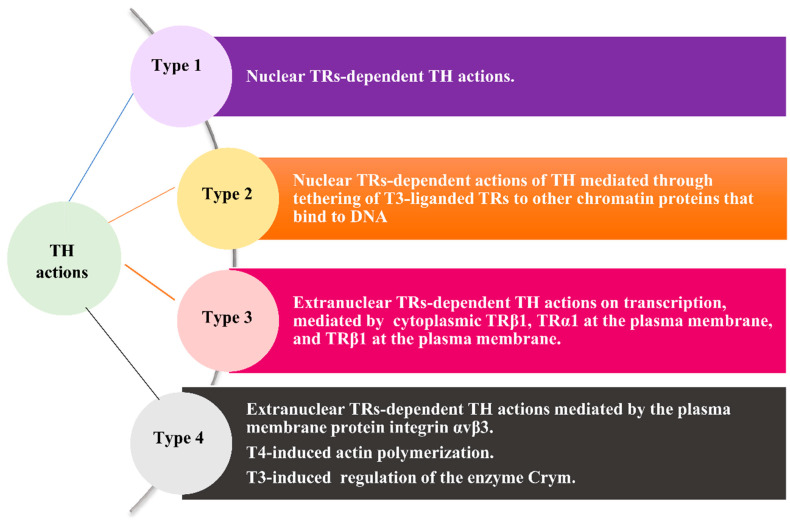
The four types of THs actions. Abbreviations: Τ3, 3,5,3′-triiodo-l-thyronine; T4, 3,3′,5,5′-tetraiodo-L-thyronine; THs, thyroid hormones; TR(s), thyroid receptor(s); TRα1, TR type alpha isoform 1; TRβ1, TR type beta isoform 1.

Type 4 mainly comprises the THs-signaling initiated at the TR located at integrin αvβ3, a member of the integrin family abundantly expressed in cancer cells and in rapidly dividing endothelial cells. The binding of T3 to THs-binding site S1 of the TR at integrin αvβ3 activates the Src/PI3K/protein kinase B (Akt) pathway, leading to a shuttling of cytoplasmic TRα to the nucleus, promoting the transcription of target genes. The binding of T4 or of T3 to THs-binding site S2 of the TR at integrin αvβ3 activates the PI3K/Akt and MAPK/ERK1/2 pathways via the activation of PLC and PKCα, leading to the phosphorylation of various nucleoproteins, including estrogen receptor type alpha (ERα) and TRβ, eventually mediating their intracellular trafficking from the cytoplasm to the nucleus [12,13]. Type 4 also includes THs actions on the polymerization of actin and the action of T3 as a regulator of Crym [37]—a metabolic enzyme playing a key role in the regulation of amino acid metabolism [36]. Figure 1 recapitulates the four types of THs actions.

The THs actions of all types coordinate to integrate the contribution of THs to the unique properties of the cancer cells designated as the “hallmarks of cancer” [37]. THs control the transcription of target genes implicated in cancer either directly or indirectly by regulating several signaling pathways positively (e.g., PI3K/Akt, endoglin, sonic hedgehog (SHH), RAS-ERK, microRNA-21) or negatively (e.g., ubiquitin-like with PHD and ring finger domains 1 (UHRF1)), or either positively or negatively (e.g., Wingless/Int (Wnt)/β-catenin). Such signaling pathways overlap in the nucleus to indirectly modulate the expression of THs target genes. THs are implicated in all hallmarks of cancer in a dual manner: promoting or inhibitory. Briefly, THs can foster cancer initiation and progression by promoting several hallmarks of cancer cells, such as: (i) proliferation (e.g., through the activation of ERK, Akt, E2F Transcription Factor 1 (EIF), p21, c-myc, mammalian target of rapamycin (mTOR), c-Fos, SHH); (ii) angiogenesis (e.g., through increased fibroblast growth factor (FGF-2), vascular endothelial growth factor (VEGF), transforming growth factor alpha (TGFa), angiopoietin 2, HIF-1α); (iii) resistance to apoptosis (e.g., through increased X-linked inhibitor of apoptosis (XIAP) as opposed to decreased BcL-2-associated X (Bax), BcLx-s, caspases, p53, FASL, (p) ERK (pERK)–cyclooxygenase (COX2) complex); (iv) invasion and migration (e.g., through increased matrix metalloproteinases (MMPs), thrombospondin 1 (TSP-1), and cathepsin H, as well as activated Src/Fak/PI3K cascade); (v) the reprogramming of metabolism, known as the Warburg effect (e.g., through the increased M2 isoform of the pyruvate kinase (PKM2)) [38]. On the contrary, THs may have an anticancer effect through inhibiting several “hallmarks of cancer cells”, such as: (i) genomic instability (e.g., through decreased Pituitary tumor-transforming gene 1 (PTTG1)) [39]; (ii) proliferation (e.g., through increased TGFb and Dickkopf 4 (DKK4), as well as decreased UHRF1); (iii) the reprogramming of metabolism (e.g., through increased Krueppel-like factor 9 (KLF9)) [40]; (iv) invasion and migration [41] (e.g., through increased DKK4, Wnt/b-catenin, and spondin 2 (Sp-2)) [42]. Additionally, THs can stimulate the apoptosis of cancer cells (e.g., through increased TNF-related apoptosis-inducing ligand (TRAIL) and decreased antiapoptotic senescence marker protein-30 (SMP30)) [37].

Intriguingly, nonclinical data indicate that THs are critical determinants of immune and inflammatory responses through the modulation of complex signaling pathways, implicating, among others, dendritic cells (DCs), T regulatory (Treg) cells, monocytes polymorphonuclear leukocytes (PMNLs), macrophages, reactive oxygen species (ROS), the NLR family pyrin domain-containing 3 (NLRP3) inflammasome, and the programmed cell death protein 1 (PD-1)/programmed cell death ligand 1 (PDL-1) interaction [14,43,44,45,46]. The net effect of THs on immune and inflammatory responses has been postulated to depend on the concentration and availability of circulating and intracellular THs and on the metabolic status that modulates NLRP3 inflammasome activation and stability [43,47]. 

The role of THs in cancer is further complicated by the fact that THs can promote senescence through the activation of ataxia-telangiectasia mutated (ATM)/adenosine monophosphate-activated protein kinase (PRKAA) proteins [37]. Senescence is a status of stable proliferative arrest triggered by various stimuli, credited with both tumor-promoting and antitumor efficacy, underpinning the biology of the many hallmarks of cancer [47,48].

**Figure 2:**
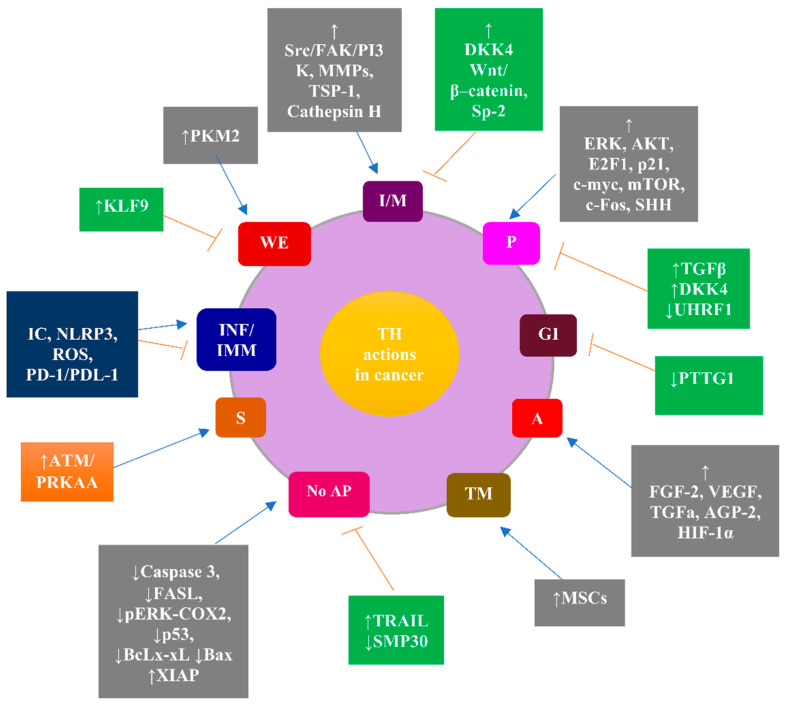
The contribution of THs to cancer hallmarks and to the senescence of cancer cells integrated by THs-modulated signaling molecules. Blue arrow: stimulation; Red arrow: inhibition. Abbreviations: A, angiogenesis; AGP-2, angiopoietin 2; AKT, protein kinase B; AP, apoptosis; ATM, activation of ataxia-telangiectasia mutated; BAX, Bcl-2-associated X protein; Bcl-Xl, B-cell lymphoma extra-large; COX2, cyclooxygenase; DKK, Dickkopf 4; E2F1, E2F transcription factor 1; ERK, extracellular signal-regulated kinases; FasL, Fas ligand; FGF2, fibroblast growth factor; GI, genomic instability; HIF-1α, hypoxia-inducible factor 1-alpha; IC, immune cells; IM, invasion/migration; INF/IMM, inflammatory/immune response; KLF9, Krueppel-like factor 9; MMPs, matrix metalloproteinases; MSCs, mesenchymal stem cells; mTOR, mammalian target of rapamycin; NLRP3, NLR family pyrin domain-containing 3; P, proliferation; PD-1, programmed cell death protein 1; PDL-1, programmed cell death ligand 1; PI3K, phosphoinositide 3-kinases; PKM2, M2 isoform of the pyruvate kinase; PRKAA, protein kinase AMP-activated catalytic subunit alpha 2; PTTG1; pituitary tumor-transforming gene 1; ROS, reactive oxygen species; S, survival; SHH, sonic hedgehog; SMP30, senescence marker protein-30; Sp-2, spondin 2; TGFa, transforming growth factor alpha; TGFβ, transforming growth factor beta; TM, tumor microenvironment; TRAIL, TNF-related apoptosis-inducing ligand; TSP-1, thrombospondin 1; UHRF1, ubiquitin-like with PHD and ring finger domains 1; VEGF, vascular endothelial growth factor 2; WE, Warburg effect; Wnt, Wingless/Int; XIAP, X-linked inhibitor of apoptosis protein.

Finally, the implication of THs in the differentiation and recruitment of mesenchymal stem cells (MSCs) in the tumor microenvironment via integrin αvβ3 builds on the versatile effects of THs on cancer progression [49]. Figure 2 illustrates the contribution of THs to the “hallmarks of cancer” and to the senescence of cancer cells mediated through various signaling molecules.

THs contribute to cancer hallmarks and to the senescence of cancer cells (depicted by the circle) by modulating several signaling molecules (depicted by the rectangles).

THs contribute to all hallmarks of cancer as follows: (i) proliferation through increased ERK, AKT, E2F1, p21, c-myc, mTOR, c-Fos, and SHH (promoting effect), and from increased TGFβ, increased DKK4, and decreased UHRF1 (inhibitory effect); (ii) genomic instability due to decreased PTTG1 (inhibitory effect); (iii) angiogenesis from increased FGF-2, VEGF, TGFa, AGP-2, and HIF-1a (promoting effect); (iv) the tumor microenvironment due to an increased recruitment and differentiation of MSCs (promoting effect); (v) the evasion of apoptosis due to decreased caspase 3, decreased FASL, decreased pERK-COX2, decreased p53, decreased BcLx-xL, decreased Bax, and increased XIAP (promoting effect), and due to increased TRAIL and decreased SMP30 (inhibitory effect); (vi) inflammatory and immune response by modulating NLRP3, ROS, and PD-1 PDL-1 (net effect either promoting or inhibitory); (vii) the reprogramming of the metabolism of cancer cells (Warburg effect) due to PKM2 (promoting effect) and KLF9 (inhibitory effect); (viii) invasion/migration due to increased Src/FAK/PI3K, MMPs, TSP-1, and cathepsin H (promoting effect), and due to increased DKK4, Wnt/β–catenin, Sp-2 (inhibitory effect); and (ix) THs promoting the senescence of cancer cells through the activation of ATM/PRKAA.

Overall, the action of THs in cancer can be promoting, integrated via signaling pathways illustrated in grey rectangle, or inhibitory, integrated via signaling pathways illustrated in green rectangles, or have a net effect with either promoting or inhibitory outcome, integrated via signaling pathways illustrated in blue rectangles. The THs-stimulated promotion of senescence integrated by ATM/ PRKAA is credited with either tumor-promoting or antitumor efficacy (orange rectangle).

## 4. Diagnosis of Hypothyroidism in Patients with Solid Non-Thyroid Cancer

To promptly diagnose hypothyroidism in patients with solid non-thyroid cancer, clinicians should be aware of its etiology, which shows similarities to and differences from that of hypothyroidism in the general population. Both in the general population and in cancer patients, defects in any step of the production or bioavailability of THs (i.e., biosynthesis, homeostasis, plasma transport, entrance, and metabolic transformations in peripheral tissues) can cause primary hypothyroidism, which is the commonest type of hypothyroidism. Defects in the hypothalamus and/or the pituitary, which control thyroid function, can cause central hypothyroidism—a less common type of hypothyroidism [2]. The most common cause of primary hypothyroidism worldwide is a deficiency of iodine in iodine-deficient geographic areas. In iodine-sufficient regions, autoimmune thyroid diseases—i.e., Hashimoto’s thyroiditis—are the leading cause of hypothyroidism. Other common causes of primary hypothyroidism include the administration of radioactive iodine to treat Graves’ disease, thyroid surgery, postpartum thyroiditis, and radiotherapy to the head or neck areas. Central hypothyroidism is caused by neoplastic, infiltrative, inflammatory, genetic, or iatrogenic disorders of the pituitary or hypothalamus.

The special causes of hypothyroidism in patients with solid non-thyroid cancer constitute an extensive issue beyond the scope of the present review. Briefly, such causes include radiotherapy, certain anticancer drugs (e.g., high dose interleukin 2 (HD IL-2), interferon type α (IFN-α), bexarotene, tyrosine kinase inhibitors (TKI), and immune checkpoint inhibitors (ICPi), mainly the anti-PD-1/PDL-1 monoclonal antibodies (mAbs)) [22,23,24,25,26,27,28,29,30,31,32,33]). The high prevalence of iodine-based contrast media (ICM)-induced hypothyroidism, reaching 15% in areas with high iodine intake [34], is a concerning issue in cancer patients who undergo numerous radiological examinations with ICM. Table 1 depicts the main, distinct causes of hypothyroidism in patients with solid non-thyroid cancer, the underlying mechanisms, and the recommended preventive measures.

To preclude the underdiagnosis of hypothyroidism of any cause, clinicians should be alert to the non-specificity and the non-sensitivity of the signs and symptoms of hypothyroidism, including impaired mental activity, weight gain, fatigue, cold intolerance, depression, weakness, dry skin, alopecia, puffiness, constipation, bradycardia, and the delayed relaxation of tendon reflexes. 

Especially in patients with solid non-thyroid cancer, hypothyroidism can be misdiagnosed as the toxicity of anticancer treatments different from thyroid toxicity, thereby leading to erroneous dose reductions or the cessation of potentially life-saving anticancer therapies. Undiagnosed hypothyroidism may lead to life-threatening myxedema coma and/or the increased toxicity of anticancer drugs due to alterations in their kinetics and clearance [23]. Therefore, the diagnosis of hypothyroidism should be based on the interpretation of suspicious clinical manifestations within a framework of measuring biochemical parameters, particularly of serum thyroid-stimulating hormone (TSH) and free (f)T4 levels. Screening tests for these biochemical parameters are conducted as indicated. Clinical or overt primary hypothyroidism is diagnosed by TSH levels above the upper reference limit and fT4 levels below the lower reference limit, while subclinical primary hypothyroidism (SCH) is diagnosed by TSH levels above the upper reference limit and fT4 levels within the normal serum range. The diagnosis of central hypothyroidism is set by fT4 levels below the lower reference limit and inappropriately low or normal TSH levels [50]. Although the recommended reference interval of TSH in most guidelines is 0.4–4 mIU/L, the attempt to harmonize the different TSH reference intervals across laboratories and countries is hampered by: (i) interindividual and intraindividual differences in thyroid parameters; (ii) age-related and ethnic variations; iii) variability in the sensitivity of different assays and in the statistical methods used to establish reference intervals [50,51].

To preclude the underdiagnosis of hypothyroidism in patients with solid non-thyroid cancer, screening tests for thyroid function are recommended before, during, and after anticancer treatment modalities related to hypothyroidism. To preclude the overdiagnosis of hypothyroidism in patients with solid non-thyroid cancer, clinicians should exclude non-thyroid illness syndrome (NTIS), as well as conditions that suppress TSH synthesis and secretion, such as the exogenous administration of glucocorticoid. Although the pathophysiology of NTIS remains elusive, it is postulated to reflect the function of hypothalamus–pituitary–thyroid axis (HPT) as a dynamic adaptive system credited with the regulation of life-history tradeoffs between reproduction, growth, immunity, and the basal metabolic rate during demanding conditions, such as cancer [52]. The biochemical parameters related to NTIS vary, mimicking either central or primary hypothyroidism [52]. Importantly, impaired HPT function in the setting of NTIS may be responsible for a difference between the interindividual physiological optimal ranges of TSH and THs levels in cancer patients compared to the laboratory-quoted normal ranges. 

Overall, close collaboration between endocrinologists and oncologists and a tailored approach are essential to promptly diagnosing hypothyroidism in patients with solid non-thyroid cancer. 

## 5. Treatment of Hypothyroidism in Patients with Solid Non-Thyroid Cancer

Originally developed in 1949, sodium salt levothyroxine (L-T4) was embraced as the mainstay of THs replacement in the late 1970s, when British endocrinologists warned against the use of desiccated thyroid, which was the first treatment for hypothyroidism. To date, more than four decades of clinical experience have consolidated the advantages of L-T4, i.e., efficacy, favorable safety profile, easy administration, good intestinal absorption, long serum half-life, and low cost [50]. The recommended starting dose of L-T4 is 1.6 μg/kg/day for adults under 65 years old with no history of cardiovascular disease, and 25–50 μg/day with titration for adults 65 years old or older and for adults with a history of cardiovascular disease [53]. L-T4 replacement needs to be tailored to establish a state of euthyroidism and to normalize the most reliable biochemical markers of adequate replacement, which are the circulating levels of TSH (and of fT4) for primary hypothyroidism and the fT4 levels for central hypothyroidism. A major challenge is to avoid both undertreatment and overtreatment of hypothyroidism. To this end, cautious titration is necessary in the case of the coadministration of drugs that interfere with L-T4 pharmacokinetics [54] or that cause spurious thyroid function tests [55]. Additionally, a combination of L-T4 plus liothyronine (L-T3) has been suggested as a potential treatment for patients with persistent hypothyroidism symptoms despite normalized thyroid function tests. However, the identification of patients with persistent hypothyroidism symptoms who may need a combination of L-T4 plus L-T3 is daunting due to the non-specificity of the symptoms of hypothyroidism. Whether the combination of L-T4 with L-T3 yields a benefit for a subset of patients is yet to be proven [56,57,58,59]. Preliminary data show that non-oncological patients with hypothyroidism who carry at least one Thr92Ala-DIO2 polymorphism allele, resulting in lower serum T3 levels, may derive clinical benefit from a combination therapy [59]. 

L-T4 replacement is indicated for patients with TSH levels > 10 mIU/L, or in the case of symptomatic patients with TSH levels between 5–10 mIU/L, measured repeatedly. SCH is most often approached with a “wait-and-see” strategy because it is a laboratory diagnosis that rarely progresses to clinically evident hypothyroidism [60,61,62,63,64,65].

Two special issues regarding the tailored treatment of hypothyroidism in patients with solid non-thyroid cancer should be considered. First, in the case of anticancer treatment-related hypothyroidism, it is critical to decide whether a cessation of the offending anticancer treatment is needed. Especially for ICPi-induced hypothyroidism, the decision-making is individualized depending on the severity of the hypothyroidism [33]. Second, the presumed, yet dual, impact of L-T4 on cancer risk raises questions about the safety of L-T4 for cancer patients. Preliminary clinical data have correlated L-T4 treatment with a significantly increased risk of lung cancer [66], as well as higher perineural invasion, a more advanced T stage according to the tumor (T)-lymph node (N)-metastasis (M) (TNM) staging system, the presence of nodal spread, and a poorer prognostic stage in pancreatic cancer [67]. Recently, a retrospective, population-based study of 601,733 cancer patients matched with 2,406,932 controls showed that L-T4 users presented with a 50% higher risk of cancer at any site (adjusted odds ratios (OR), 1.50, 95% CI: 1.46–1.54; *p* < 0.0001) compared to nonusers. The increase in risk was also significant in terms of brain cancer (adjusted (a) odds ratio (OR) (aOR), 1.90, 95% confidence interval (CI), 1.48–2.44; *p* < 0.0001), skin cancer (aOR, 1.42, 95% CI, 1.17–1.72; *p* < 0.0001), pancreatic cancer (aOR, 1.27, 95% CI, 1.01–1.60; *p* = 0.03), and female breast cancer (aOR, 1.24, 95% CI, 1.15–1.33; *p* < 0.0001) [68]. On the other hand, L-T4 treatment has been correlated with a decreased risk of colorectal cancer [69,70,71]. 

Overall, close collaboration between endocrinologists and oncologists is essential to tailor the treatment of hypothyroidism in patients with solid non-thyroid cancer. 

## 6. Clinical Data on Hypothyroidism as a Potential Predictive Factor for Solid Non-Thyroid Cancer

The currently available clinical data concerning hypothyroidism as a potential predictor of the risk of solid non-thyroid cancer are variable and conflicting. 

### 6.1. Clinical Data Indicating an Association between Hypothyroidism or TSH Levels near the Upper Normal Range and a Decreased Risk of Solid Non-Thyroid Cancer

A nationwide cohort study enrolling 61,873 women with hypothyroidism demonstrated a decreased incidence of breast cancer in women with hypothyroidism compared to the general population (standardized incidence ratio (SIR), 0.94, 95% CI: 0.88–1.00). This association was not modified after patient stratification by cancer stage at diagnosis, estrogen receptor (ER) status, age, comorbidity, history of alcohol-related disease, and obesity [72]. 

A cohort study of 62,546 Korean women, aged 40 years old or older without breast cancer at baseline, followed for 4.8 years (interquartile range: 2.8–7.3 years), showed that TSH levels in the highest tertile within the euthyroid range were associated with a lower risk of breast cancer than TSH levels in the lowest tertile (adjusted (a) hazard ratio (HR) (aHR), 0.68; 95% confidence interval (CI), 0.55–0.84) [73]. 

The largest to date metanalysis investigating the correlation between thyroid diseases (both benign and malignant) and the risk of breast cancer involved 67,049 female patients with breast cancer and revealed an association between hypothyroidism and a reduced risk of breast cancer with no heterogeneity (OR, 0.95; 95% CI: 0.91–1.00, *p* = 0.042; I^2^ = 0.0%) contrary to the significant association of an increased risk of breast cancer with hyperthyroidism [74]. 

A prospective cohort study of 134,122 multiethnic US postmenopausal women, aged 50–79 years old, showed a significant inverse association between invasive breast cancer and a history of hypothyroidism (HR, 0.91; 95% CI, 0.86–0.97) [75].

A prospective cohort study on a community-dwelling population, aged 25–84 years old, in Western Australia detected, during a 20-year follow-up, 600 cases of newly diagnosed nonskin cancer, in particular, 126 cases of prostate cancer, 100 cases of breast cancer, 103 cases of colorectal cancer, and 41 cases of lung cancer. Higher TSH levels were associated with a lower risk of prostate cancer after adjusting for potential confounders, with a 30% lower risk for every 1 IU/L increase in TSH (aHR: 0.70, 95% CI, 0.55–0.90, *p* = 0.005) [76]. 

In a prospective study on 402 prostate cancer patients and 800 controls, hypothyroidism was correlated with a lower prostate cancer risk compared to euthyroidism (OR, 0.48). TSH in the highest quintiles (Q5) was associated with a decreased risk of cancer compared to the lower quintiles (Q1–4) (Q5 versus Q1–4: OR, 0.7) [77]. 

Among 1314 patients with incident endometrial cancer (EC) derived from the Nurses’ Health Study (NHS), conducted from 1976 to 2010, and from an NHSII study, conducted from 1989 to 2011, a history of hypothyroidism of 8 years or more (median duration) was inversely associated with EC (relative risk (RR), 0.81; 95% CI, 0.63–1.04; *p*-trend with history duration = 0.11) [78]. 

In a nationwide, population-based case–control study on 139,426 patients, conducted in 2018 in Taiwan to assess the association between thyroid disorders and colorectal cancer (CRC) risk, hypothyroidism in all age groups was correlated with a 22% lower risk of CRC (OR, 0.78; 95% CI, 0.65–0.94; *p* = 0.008) and a 45% lower risk of rectal cancer (OR, 0.55; 95% CI, 0.40–0.76; *p* < 0.001) compared to controls. Patient stratification by age group revealed that the decrease in CRC risk related to hypothyroidism was statistically significant only for patients with a rectal cancer diagnosis aged 50 years old or older (aOR, 0.54; 95% CI, 0.39–0.74; *p* < 0.001) [79]. 

A metanalysis of 19 studies assessing the relationship between hypothyroidism and breast cancer risk, including 6 cohort studies and 13 case–control studies, revealed that hypothyroidism was associated with a lower risk of breast cancer in the European subgroup (OR, 0.93; 95% CI, 0.88–0.99) [80].

### 6.2. Clinical Data Indicating an Association between Hypothyroidism and an Increased Risk of Solid Non-Thyroid Cancer

Untreated hypothyroidism has been associated with an increased risk of CRC (adjusted OR, 1.16; 95% CI, 1.08–1.24, *p* < 0.001) in a large case–control study comparing 20,990 CRC patients with 82,054 matched controls, followed for an average period of 6.5 years [69]. 

Higher TSH levels were associated with increased CRC incidence in a prospective cohort study involving community-dwelling men, aged 70–89 years old, with a follow-up period of 9 years (subhazard ratio (SHR), 1.19; 95% CI 1.00–1.42; *p* = 0.048 for every 1 standard deviation (SD) increase in log TSH). This association was diminished after excluding men with SCH from the analysis (SHR, 1.14; 95% CI, 0.87–1.49; *p* = 0.339). The association between higher TSH levels and increased CRC incidence was nonsignificant after comparing men with SCH to a euthyroid referent category (SHR, 1.56; 95% CI, 0.92–2.63; *p* = 0.096). The association of higher TSH levels with increased CRC incidence remained significant after excluding the first year of follow-up (SHR, 1.23; 95% CI, 1.02–1.48, *p* = 0.028) [81].

In a case–control study on 273 cases of colorectal neoplasm, SCH was indicated as an independent risk factor for colorectal neoplasm (OR, 1.689, 95% CI: 1.207–2.362; *p* = 0.002), after adjusting for blood pressure, body mass index (BMI), hypertension, and smoking [82]. 

Hypothyroidism has been correlated with an increased risk of hepatocellular cancer (HCC) as well. A case–control study enrolling 420 patients with HCC and 1104 healthy controls demonstrated a significantly higher risk of HCC in women with hypothyroidism of a duration longer than a decade, independent of established HCC risk factors (OR, 2.9 following regression analysis for risk factors), but the thyroid status was evaluated by patient self-reporting using questionnaires without tests of thyroid function [83].

Hypothyroidism has been suggested to be a potential risk factor for liver carcinogenesis, being more common in HCC with no recognized etiology compared to HCC with HCV (aOR, 12.7; 95% CI, 1.4–117.1) and compared to all controls (aOR, 6.8; 95% CI, 1.1–42.1) [84]. In fact, hypothyroidism has been increasingly [85,86], though not consistently [87,88], correlated with nonalcoholic fatty liver disease (NAFLD), which encompasses a wide range of liver disorders extending from steatosis/nonalcoholic fatty liver disease (NAFLD) to nonalcoholic steatohepatitis (NASH), cirrhosis, and, eventually, HCC [89]. However, whether hypothyroidism is the link between NAFLD and HCC remains unknown. 

A population-based cohort study on 115,746 participants without a history of thyroid disease, aged 20 years old or over, recruited from four nationwide health screening centers in Taiwan from 1998 to 1999, assessed the association between cancer risk and SCH, which, in this study, was defined as a TSH level of 5.0–19.96 mIU/L with normal total T4 (TT4) levels. The authors demonstrated that SCH was correlated with an increased risk of breast cancer, bone cancer, or skin cancer compared to euthyroidism (RR, 2.79; 95% CI, 1.01–7.70) [90]. 

### 6.3. Clinical Data Indicating the Opposing Effects of THs on the Risk of Solid Non-Thyroid Cancer or No Significant Association between Hypothyroidism and the Risk of Solid Non-Thyroid Cancer

A recent study of 375,635 Israeli patients with no prior history of cancer that evaluated the correlation between TSH and fT4 levels and overall cancer risk, as well as with the risk of specific cancer types, revealed the opposing effects of THs on cancer risk. In this study, the effect of THs on cancer risk depended on the age of the patient and the type of cancer. Based on the Israel National Cancer Registry, 23,808 cases of cancer were detected over a median follow up of 10.9 years. Among patients aged less than 50 years old at inclusion, TSH levels in the hyperthyroid range, elevated fT4 levels, and subclinical hyperthyroidism were associated with an increased cancer risk (HR, 1.3, 1.28, and 1.31, respectively). Increased TSH levels were correlated with a decreased risk of prostate cancer (HR, 0.67), while log-TSH elevation was associated with an increased risk of melanoma (HR, 1.11) and uterine cancer (HR, 1.27) [91]. 

A metanalysis of 12 population-based studies evaluating the association of thyroid dysfunction with the risk of breast cancer, including 24,571 cases, demonstrated no significant correlation between hypothyroidism and breast cancer (OR, 0.83; 95% CI, 0.64–1.08, *p* = 0.162) [92].

A metanalysis of 19 studies, including 6 cohort studies and 13 case–control studies, demonstrated no correlation between hypothyroidism and the risk of breast cancer (OR, 0.90; 95% CI, 0.77–1.03) worldwide [80].

A prospective cohort study on a community-dwelling population, aged 25–84 years old, in Western Australia revealed an absence of association between TSH levels, fT4 levels, or antibodies against thyroid peroxidase (anti-TPO Abs) and all nonskin cancer events (i.e., prostate, breast, colorectal, and lung cancers combined), or with breast, colorectal, or lung cancer [75]. 

## 7. Clinical Data on Hypothyroidism as a Potential Prognostic Factor for Solid Non-Thyroid Cancer

The currently available data on the association between hypothyroidism and solid non-thyroid cancer prognosis are variable and conflicting. 

### 7.1. Clinical Data Indicating an Association between Hypothyroidism and a Favorable Prognosis for Solid Non-Thyroid Cancer

The hallmark case report on the remission of metastatic NSCLC ascribed to amiodarone-induced myxedema coma by Hercbergs and Leith in 1993 [35] launched an evolving field of clinical research on the anticancer potential of hypothyroidism. In 2010, a review of the published up-to-that-time clinical studies indicated that hypothyroidism of any cause—i.e., spontaneous or iatrogenic—is an omen of favorable cancer prognosis thanks to inhibition of the “permissive role of THs” in cancer [93]. Since then, accumulating data have consolidated the association of hypothyroidism or hypothyroxinemia with the favorable prognosis of solid non-thyroid cancer, strengthening the hypothesis of the anticancer potential of hypothyroidism. 

In a study of HCC patients, univariate analysis indicated a significant association between fT4 levels at the time of HCC diagnosis lower than or equal to 1.66 ng/dL and improved median OS compared to fT4 levels at the time of HCC diagnosis higher than 1.66 ng/dL: 10.6 months (95% CI, 7.5–13.6 months) versus 3.3 months (95% CI, 2.2–4.3 months), respectively; *p* = 0.007 [94].

A retrospective case control study assessing the occurrence of primary hypothyroidism among 1979 patients with NSCLC and small-cell lung cancer (SCLC) (stages I–IV) demonstrated that the number of patients with hypothyroidism differed among distinct disease stages, with 86 patients, 46 patients, 19 patients, and 19 patients diagnosed with disease stages III, IV, I, and II, respectively. Disease stages I–IV showed improved median survival in hypothyroid patients compared to euthyroid patients (14.5 months versus 11.1 months, respectively; *p* = 0.014). In particular, hypothyroid patients in stage IV of the disease experienced a median survival of 11 months, significantly higher than that of euthyroid patients in stage IV (5 months) (*p* = 0.0018). Overall, in this study, hypothyroidism was suggested to be a significant prognostic factor for a favorable clinical outcome with respect to lung cancer [95].

Iatrogenic hypothyroidism has been shown to lead to favorable cancer prognosis, either via increasing the response to standard anticancer treatment or by itself. In a patient with a high-grade optic glioma, hypothyroidism induced by antithyroid drugs, in conjunction with carboplatin, led to a rapid response to carboplatin, tumor regression within 4 weeks, an extended remission period (2.5 years), and a prolonged OS (4.5 years) [96]. This effect was consistent with the results of a previous phase I/II study on 22 patients with recurrent glioma, according to which, propylthiouracil-induced hypothyroidism followed by tamoxifen led to significantly longer median survival compared to euthyroidism (10.1 months versus 3.1 months, respectively; *p* = 0.03) [97]. In an uncontrolled observational study on 23 patients with end-stage solid tumors (brain, ovary, lung, pancreas, salivary gland, breast cancer, mesothelioma, soft-tissue sarcoma), inducing euthyroid hypothyroxinemia via the administration of methimazole and L-T3, or the replacement of L-T4 with L-T3 in patients with preexisting primary hypothyroidism, led to prolonged survival in 83% of patients, exceeding the 1-year survival estimated by the SEER and AJCC databases, as well as literature reports (20%) [98]. Rodríguez-Molinero et al. reported two cases of biochemical response in tumors due to treatment with antithyroid drugs and the exogenous administration of L-T3, one in a female patient with metastatic triple negative breast cancer and the other in a female patient with metastatic pancreatic cancer. The second patient also experienced regression in a cutaneous metastasis. The response was attributed to exogenous L-T3, since neither of the patients were treated with an anticancer treatment. An inverse relationship between serum fT3 levels and the levels of the corresponding tumor markers (Ca 125 for breast cancer and Ca 19.9 for pancreatic cancer) was observed. However, in both cases, tumor progression and an increase in serological markers occurred concomitantly with a decline in plasma fT3 levels, despite an increase in exogenous L-T3 administration. The administration of LT3 in the first patient, and methimazole in the second patient, required cessation due to adverse events. Interestingly, the authors postulated that the decline of T3 in plasma could be ascribed to an excess of consumption or inactivation caused by the tumor; thus, it could represent a novel biomarker of tumor progression [99]. 

A prospective study on 333 women with EC, followed for a median period of 35 months, demonstrated that a clinical history of hypothyroidism (TSH > 4.5 mIU/L) was associated with improved survival outcomes (HR, 0.34, 95% CI, 0.11–1.10), though not significantly in the unadjusted model (*p* = 0.07). A history of hypothyroidism (TSH > 4.5 mIU/L) was associated with a 78% reduced risk of death (adjusted HR, 0.22; 95% CI, 0.06–0.74; *p* = 0.02) compared to euthyroidism after adjusting for age, BMI, socioeconomic deprivation quintile, type 2 diabetes mellitus, histological subtype, lymphovascular invasion, depth of myometrial invasion, stage, and grade of endometrial cancer. Hypothyroidism was also associated with improved cancer-specific survival (adjusted HR, 0.21, 95% CI; 0.05–0.98; *p* = 0.04) and fewer recurrences (adjusted HR, 0.17; 95% CI, 0.04–0.77; *p* = 0.02) compared to euthyroidism. SCH was associated with a 69% reduced risk of death (HR, 0.31; 95% CI, 0.09–1.28) compared to euthyroidism. After adjustment for confounding factors, SCH was associated with a 46% reduced risk of death (HR, 0.54; 95% CI, 0.12–2.43) compared to euthyroid women [100]. 

A study on a discovery cohort of 1692 patients with newly diagnosed solid cancer brain metastases (BMs) treated at the Medical University of Vienna, and an independent validation cohort of 191 patients with newly diagnosed BMs treated at the University Hospital Zurich, indicated hypothyroidism is an independent prognostic factor of favorable prognosis. In the discovery cohort, hypothyroidism was significantly associated with an improved survival prognosis related to the diagnosis of cancer (31 months versus 21 months; *p* = 0.0026) and with an improved survival prognosis related to the diagnosis of BMs (12 months versus 7 months; *p* = 0.0079). In this study, normal ranges were 0.44–3.77 mIU/L for TSH, 2.15–4.12 pg/mL for fT3, 0.76–1.66 ng/dL for fT4, 0.8–1.8 ng/mL for T3 and 58–124 ng/mL for T4. In a multivariate analysis, including the diagnosis-specific graded prognostic assessment score, the primary tumor type, and sex, hypothyroidism was an independent factor associated with improved survival related to the diagnosis of BMs (HR, 0.76; 95% CI, 0.63–0.91; *p* = 0.0034). The validation cohort consolidated the association between hypothyroidism and improved survival prognosis related to the diagnosis of cancer (55 months versus 11 months; *p* = 0.00058), as well as related to the diagnosis of BMs (40 months versus 10 months; *p* = 0.0036) [101]. 

Recently, Hou et al. reported a case of a hypothyroid 71-year-old man with stage IV lung adenocarcinoma who, until the last follow-up, experienced survival longer than the median survival of lung adenocarcinoma (2.5 years versus 4–13 months, respectively). Τhe patient did not receive any THs replacement despite being diagnosed with severe hypothyroidism, with fT3 = 1.71 pmol/L (normal range, 3.09–7.42 pmol/L), f T4 = 0.86 pmol/L (normal range, 7.64–16.03 pmol/L), and TSH = 62.21 mIU/L (0.49–4.91 mIU/L) accompanied by a myxedematous face and an enlargement of the thyroid found in chest computed tomography (CT). Given that the patient received no anticancer treatment, the authors attributed the prolongation of his survival to hypothyroidism. Repeated chest CT scans revealed decelerated, but not arrested, tumor growth [102]. 

Table 2 summarizes the abovementioned clinical data, indicating an association between hypothyroxinemia or hypothyroidism and the favorable prognosis of solid non-thyroid cancer.

### 7.2. Clinical Data Indicating an Association between Hypothyroidism or Decreased THs Levels and an Unfavorable Prognosis of Solid Non-Thyroid Cancer

In a large prospective cohort study on 212,456 middle-aged South Korean euthyroid people, after a median follow-up of 4.3 years, fT3 levels within the euthyroid range were inversely associated with cancer mortality (HR, 0.62; 95% CI, 0.45–0.85; *p* for linear trend = 0.001). In particular, both fT3 and fT4 levels were inversely associated with liver cancer mortality (HR per SD change: 0.64 for fT3, 0.52 for fT4) [103].

An Austrian multicenter trial enrolling 199 patients with EC indicated that TSH > 2.5 mIU/L before the initiation of anticancer treatment was associated with significantly worse 5-year disease-free and disease-specific survival compared to TSH ≤ 2.5 mIU/L in the univariate analysis. In the multivariable analysis, TSH > 2.5 mIU/L was independently associated with significantly worse 5-year disease-specific survival compared to TSH ≤ 2.5 mIU/L (HR, 2.7; 95% CI, 1.1–6.7; *p* = 0.03) [104]. 

In a study on 838 patients diagnosed with nonsurgical HCC, elevated TSH levels were associated with larger tumors. Moreover, higher TSH levels demonstrated a significant association with worse outcome in the univariate analysis, with a median OS of 12.3 months (95% CI, 8.9–15.7 months) for TSH ≤ 1.7 mIU/L versus 7.3 months (95% CI, 5.4–9.2 months) for TSH > 1.7 mIU/L (*p* = 0.003). However, this effect was not confirmed in the multivariate analysis after adjustment for other prognostic factors, including Child–Pugh class, tumor size, performance status, macrovascular invasion, extrahepatic spread, tumor treatment, α-fetoprotein (AFP), and C-reactive protein (CRP) levels [94].

A novel model for predicting the progression of HCC that incorporates TSH levels was recently suggested by Yu et al. The authors studied a retrospective (*n* = 1005) and a prospective (*n* = 77) cohort with HCC to develop and validate the efficacy of a novel prognostic model leveraging TSH levels and three more variables (Barcelona Clinic Liver Cancer (BCLC) stage, the presence of portal vein tumor thrombus, an alpha-fetoprotein level). This model proved superior in predicting progression-free survival (PFS) over conventional tumor scoring systems, such as the Child–Pugh score, Model for End-stage Liver Disease (MELD), TNM staging system, Okuda classification, and CLIP score. In the prospective cohort used to validate the correlation between TSH levels and tumor progression in patients with HCC, the KM curve indicated a shorter PFS in the high TSH level group compared to the low TSH level group (*p* = 0.001) [105].

The first study to assess the association between SCH and cancer mortality enrolled 115,746 adult Taiwanese, of whom 1841 presented with SCH (TSH: 5.0–19.96 mIU/L) and 113,905 presented with normal thyroid function (TSH: 0.47–4.9 mIU/L). During a follow-up period of 1,034,082 person-years, 1532 cancer-related deaths were reported. After adjusting for age, gender, BMI, diabetes mellitus, hypertension, dyslipidemia, smoking, alcohol drinking, betel nut chewing, physical activity, income, and education level, the relative risk (RR) of cancer-related deaths in subjects with SCH versus euthyroid subjects was 1.51 (95% CI, 1.06–2.15). Cancer site analysis revealed a significantly increased risk of bone, skin, and breast cancer among SCH subjects (RR, 2.79; 95% CI, 1.01–7.70). More profound risks of total cancer-related deaths were reported for older patients (RR, 1.71; 95% CI, 1.02–2.87), females (RR, 1.69; 95% CI, 1.08–2.65), and heavy smokers (RR, 2.24; 95% CI, 1.19–4.21) [90].

In a case–control study of 273 cases of colorectal neoplasm, SCH, compared to euthyroidism, was significantly correlated with more advanced colonic lesions (*p* = 0.028) and colorectal cancer (*p* = 0.036) [82]. 

In a recent systematic review addressing the impact of hypothyroidism on the risk of cancer incidence and cancer mortality [106], two studies showed increased cancer mortality among patients with SCH compared to euthyroid individuals [90,94]. 

Table 3 summarizes the main clinical data indicating hypothyroidism or decreased THs, even within the euthyroid range, as predictors of unfavorable prognoses with respect to solid non-thyroid cancer. 

### 7.3. Clinical Data Indicating the Absence of a Significant Association between Hypothyroidism, THs Levels, or TSH Levels and the Prognosis of Solid Non-Thyroid Cancer

Data on the baseline thyroid function of 1587 participants from the Osteoporotic Fractures in Men (MrOS) study—a cohort of community-dwelling US men aged 65 years old or older—were analyzed to assess the association of thyroid function with mortality. Over a mean follow-up of 8.3 years, the fully adjusted models showed, among other findings, no association between baseline TSH levels and cancer death (relative hazard (RH), 0.96 per mIU/L; 95% CI, 0.85–1.07) [107]. 

In a large prospective cohort study on 212,456 middle-aged South Korean euthyroid people, after a median follow-up of 4.3 years, TSH levels showed no association with mortality endpoints [103].

A prospective study assessed the association between hypothyroidism and cancer-related mortality in a cohort of 75,076 women, aged 20–89 years old and certified as radiologic technologists in the United States, from 1926 to 1982. The medical history of these women was ascertained through self-completed questionnaires during the 1983–1998 period. All women reported no malignant disease or benign thyroid disease apart from thyroid dysfunction. This cohort was followed through the Social Security Administration database and the National Death Index Plus. Within a follow-up period equal to or longer than 10 years, the hypothyroid women showed no increase in cancer-related mortality [108]. 

The first prospective study that demonstrated a significant association between increased T3 levels and the aggressiveness of breast cancer in the Malmö Preventive Project also showed no statistically significant associations between T3 levels and deaths due to other cancer types (age-adjusted HR, 1.09; 95% CI, 0.72–1.65) or all-cause mortality (HR, 1.25; 95% CI, 0.97–1.60) [109]. 

A prospective cohort study on 134,122 multiethnic postmenopausal women in the US, aged 50 to 79 years old, showed no significant impact from THs status on Surveillance, Epidemiology, and End Results (SEER) stages, histologic types, morphologic grades, ER status, PR status, or the human epidermal growth factor receptor 2 (HER2) status of breast cancer [75]. 

In a retrospective cohort study on breast cancer patients, increasing serum TSH levels showed no significant correlation with tumor grade, overall stage according to American Joint Committee on Cancer (AJCC), tumor size, lymph node positivity, or the presence of metastasis. All time-weighted and unweighted median TSH levels were within normal reference ranges [110]. 

The first study to distinguish between the impact of prevalent hypothyroidism (i.e., present at the time of the breast cancer diagnosis) and incident hypothyroidism (i.e., diagnosed during follow-up) on breast cancer outcomes was a population-based cohort study in Denmark, including 35,463 women with breast cancer followed for 212,641 person-years. In this study, neither prevalent nor incident hypothyroidism were associated with breast cancer recurrence (adjusted HR for prevalent hypothyroidism, 1.01; 95% CI 0.87–1.19; adjusted HR for incident hypothyroidism, 0.93; 95% CI, 0.75–1.16). These results were confirmed after stratification by menopausal status, ER status, chemotherapy, or radiotherapy [111]. 

In a recent systematic review [106], one study showed no association between SCH and cancer mortality among men aged 65 years old or older [107].

Table 4 summarizes the clinical data indicating no significant association between hypothyroidism, THs levels, or TSH levels and cancer prognosis.

## 8. Clinical Data Indicating an Association between Hypothyroidism Induced by Anticancer Treatment and Favorable Prognoses

Hypothyroidism induced by anticancer treatment has been increasingly [112,113,114,115,116,117,118,119,120,121,122], though not consistently [123], correlated with improved clinical outcomes of solid non-thyroid cancer, but a detailed discussion of this issue is beyond the scope of the present review. Herein, we provide some relevant representative data. 

In a phase-III trial comparing two protocols comprising a combination of cisplatin chemotherapy and radiotherapy in 300 patients with locally advanced head and neck cancer, hypothyroidism was correlated with a lower locoregional failure rate (LRFR) (HR, 0.342, *p* = 0.043) and a longer OS (HR, 0.336, *p* = 0.001). A longer duration of hypothyroidism and TSH levels up to 40 mIU/L were correlated with lower LRFR and longer PFS and OS [112]. 

Though considered a side effect of therapy, ICPi-induced hypothyroidism and TKI-induced hypothyroidism may be correlated with improved clinical outcomes, but not consistently. It is acknowledged that most relevant studies assess thyroid disorders collectively, but hypothyroidism is the most common thyroid disorder related to ICPi and TKI. Of 51 patients with advanced non-small-cell lung cancer (NSCLC) treated with pembrolizumab (anti-PD-1 monoclonal antibody (mAb)) as part of the KEYNOTE-001 study (NCT01295827), the patients who developed thyroid dysfunction experienced significantly longer overall survival compared to those without thyroid dysfunction [113]. In another study, among 194 NSCLC patients treated with nivolumab (anti-PD-1 mAb), thyroid dysfunction related to treatment was indicated to be an independent predictive factor of longer OS and PFS compared to an absence of thyroid dysfunction [114]. In another study of 111 patients with NSCLC treated with nivolumab, low fT4 levels induced by nivolumab in NSCLC patients were significantly correlated with longer PFS and longer median OS compared to normal fT4 levels [115]. 

Interestingly, a retrospective analysis of data from patients with advanced solid tumors treated with anti-PD-1 mAbs (nivolumab, pembrolizumab) indicated the previous treatment with TKI was a major risk factor for immune-related hypothyroidism [116]. 

Nevertheless, many unresolved issues concerning the association between immune-related thyroid disorders and the efficacy of ICPi have emerged, as discussed by the authors of the present review elsewhere [123]. A causative link connecting immune-related hypothyroidism with the therapeutic efficacy of ICPi is yet to be identified [117]. 

A metanalysis revealed that, among patients with metastatic renal cell carcinoma (mRCC) treated with sunitinib, the patients who developed related hypothyroidism showed significantly improved OS and non-significantly improved PFS compared to patients without hypothyroidism [118]. Hypothyroidism related to treatment with TKI is constantly reported to provide a clinical benefit for TKI-treated cancer patients [119,120,121]. A high baseline fT3/fT4 ratio is strongly associated with improved clinical outcomes in patients with progressive metastatic CRC treated with regorafenib [122].

According to PROCLAIΜ—a registry of patients receiving HD IL-2—the development of thyroid dysfunction was correlated with improved tumor control and OS [123]. 

## 9. Harnessing Hypothyroidism in the Treatment of Solid Non-Thyroid Cancer: Current Challenges and Future Perspectives for a Personalized Approach

The wealth of data on the role of hypothyroidism in the incidence and prognosis of solid non-thyroid cancer generates a pending query of paramount clinical importance: Can hypothyroidism—or THs replacement—be harnessed as an anticancer modality for patients with solid non-thyroid cancer? Indeed, the hypothesis of harnessing hypothyroidism as an anticancer strategy is rationalized by reports of an association between hypothyroidism and decreased risk and favorable clinical outcomes with respect to solid non-thyroid cancer, while the hypothesis of harnessing THs replacement as an anticancer strategy is rationalized by reports an association between hypothyroidism and increased risk and unfavorable cancer outcomes. Nevertheless, whether and how to harness hypothyroidism or THs replacement in cancer therapeutics remains uncertain. The present review suggests four prerequisites for a personalized approach to this issue.

The first prerequisite is to untangle the dual role of THs in cancer. It has been speculated that the transduction of THs-induced signaling in the setting of cancer is dependent on the type of cancer and on the molecular context, but the underlying mechanisms have yet to be elucidated.

Preliminary data have indicated certain determinants that tilt the swinging pendulum of the THs-induced signaling toward the promotion or inhibition of cancer, which await validation. For instance, the nuclear and cytoplasmic forms of TRβ1 have been shown to exert opposite roles in breast tumorigenesis. In a well-characterized cohort of 274 primary breast cancer patients, the nuclear TRβ1 was indicated to be an independent and adverse prognostic marker, whereas the cytoplasmic TRβ1 was indicated to be an independent and favorable prognostic marker [124]. Additionally, nonclinical data have indicated the tumor suppressive role of TRβ1 in the setting of HCC [125] as opposed to the prometastatic role of TRα1 in this cancer type [126]. Moreover, TRα2 expression has been associated with improved disease-free survival (DFS) in multifocal/multicentric breast cancer, contrary to the association of TRα1 with unfavorable DFS in unifocal breast cancer [127]. Furthermore, the association between hypothyroidism and cancer risk may show a genetical predisposition, as indicated by the reported association between hypothyroidism and increased breast cancer risk in patients harboring the single-nucleotide polymorphism (SNP) rs 2235544 in the DIO1 gene [128]. Interestingly, it has also been speculated that oncogenic mutations affecting different cellular targets of THs may be responsible for the dual actions of THs in cancer. For instance, mutations that affect the balance between the degradation and stabilization of β-catenin have been implicated in the cell-specific effect of THs on cancer cell growth [129]. 

Four major hurdles to understanding the dual role of THs in cancer need to be overcome. First, it is essential to precisely assess the intratissue and the intracellular status of THs, which may not be represented by the serum levels of TSH and/or THs. Ongoing efforts to define the metabolome signature of THs are anticipated to accurately assess the intratissue and intracellular status of THs [130,131]. Second, it is important to clarify whether thyroid autoimmunity and/or aberrant dietary iodine intake—well-established pathogenetic mechanisms of hypothyroidism—may precipitate higher cancer incidence and/or more aggressive cancer behavior. For instance, further prospective studies are required to explore a potential causality in the emerging association of breast cancer in the premenopausal setting with autoimmune thyroid disorders [132] or iodine deficiency [133]. Third, it has been hypothesized that hypothyroidism may promote cancer initiation and progression indirectly by interfering with established cancer risk factors, such as the metabolic syndrome, which is significantly associated with endometrial, pancreatic, breast (postmenopausal), rectal, and colorectal cancer [134,135]. More efforts to consolidate a pathogenetic link between thyroid function and cancer could counteract the second and the third hurdle. Fourth, clinicians should bear in mind that the association between TSH and fT4 may not be straightforward in cancer patients due to alterations in the HPT axis caused by malnutrition, elevated endogenous cortisol levels, the exogenous administration of glucocorticoids, or NTIS. In those cases, further research could clarify whether it is necessary to revise the reference ranges of TSH and THs levels, especially for cancer patients.

The second prerequisite is to devise strategies to abrogate the tumor-promoting effects of THs and/or mimic the anticancer effects of THs. These strategies could include interventions in THs status or the development of new pharmaceutical agents. Examples of such interventions are: (i) the induction of euthyroid hypothyroxinemia via antithyroid drugs and the concomitant administration of L-T3 [98,99] and (ii) the administration of L-T3 instead of L-T4 as a THs replacement. These interventions capitalize on the knowledge that T4 is responsible for most of the tumor-promoting effects of THs, while T3 may exert an anticancer effect [98,136,137].

Interestingly, it has been suggested that L-T3 could be repurposed as an immunotherapeutic agent in cancer therapeutics. Indeed, L-T3 has been shown in vitro and in vivo to enhance anticancer immune surveillance in a broad spectrum of cancers, particularly in CRC, by: (i) unleashing the immune surveillance that was suppressed by TIGIT/PVR interaction; (ii) reversing the inhibition of IL-2 secretion by Jurkat cells and reinforcing the function of T cells in the activated PBMCs; (iii) increasing the quantity and the quality of tumor-infiltrating CD8^+^ T cells [138]. 

Given that the TIGIT/PVR interaction synergizes with the PD-1/PD-L interaction [139], a synergistic anticancer effect between the L-T3-mediated blockade of TIGIT/PVR and the PD-1/PD-L1 blockade merits further evaluation. This perspective is clinically important considering that small molecular immune modulators with anticancer efficacy are expected to revolutionize cancer immunotherapy [140]. Moreover, vaccination with T3-stimulated DCs has shown an anticancer efficacy in mice. If this observation is confirmed in humans, T3 could potentiate a promising strategy of DC-based anticancer vaccine [141]. However, the use of conventional L-T3 preparations is hampered by difficulties in monitoring serum T3 levels, interpreting TSH levels, and titrating L-T3 doses. To counteract these difficulties, a slow-release L-T3 preparation is required [57].

On the other hand, nonclinical and clinical data show a potential role of T3 in cancer progression through several mechanisms, which challenge the endorsement of L-T3 as an anticancer strategy [126,142,143,144]. 

Tetrac—a deaminated form of T4—or its nanoparticulated analogue, nano-diamino-tetrac (NDAT), could exert an anticancer effect. In preclinical settings, tetrac and NDAT have been recognized as anticancer agents that antagonize the binding of THs to TRs at integrin αvβ3, thereby inhibiting tumor-promoting THs signaling. The anticancer actions of tetrac include: (i) the abrogation of cancer cell proliferation, angiogenesis, and metastasis; (ii) the stimulation of cancer cell apoptosis; (iii) the enhancement of immune surveillance; (iv) the repair of double-strand DNA breaks; (v) chemosensitization; (vi) radiosensitization [13,145,146,147,148,149,150]; and (vii) modulating the transcription of cancer cell genes independently of T4 and T3 [151]. The functions of tetrac and NDAT in the clinical setting have yet to be elucidated.

An additional modality to attenuate the tumor-promoting effect of THs could be the development of pharmaceutical agents to selectively block the TSH/TSH receptor (TSHR) interaction in cancer cells, which is known to transduce a tumor-promoting signal [152,153].

A major challenge for the pharmaceutical industry is the development of effective and safe thyromimetics with anticancer properties that uncouple the therapeutic actions of THs from the detrimental effects of a thyrotoxic state. The first two TRβ-selective thyromimetics with multiple therapeutic applications, including anticancer, that entered clinical trials were Sobetirome (GC-1) and the eprotirome (KB2115); however, these agents were withdrawn early due to toxicity [154]. The future design of “tissue-selective prodrugs” may provide the active compound at the site of action [155].

Moreover, improved understanding of the emerging clinical implications of endogenous THs metabolites may pave the way for the development of THs replacement regimens that act concomitantly as anticancer strategies [156]. 

The third prerequisite is the appropriate selection of patients who may derive benefit. To this end, it is necessary to continue basic and translational research on the molecular background of THs signaling, pursuing its interaction with well-established oncogenic molecular pathways. Hopefully, such research will yield valid tumor-specific and patient-specific biomarkers to identify patients with tumors sensitive to THs and predict the impact of THs on cancer outcomes. 

The fourth prerequisite is to counteract current methodological limitations, including: (i) the scarcity of data on the distinction between incident and prevalent hypothyroidism; (ii) the scarcity of data on distinction between hypothyroxinemia and hypothyroidism; (iii) the absence of tests for thyroid function in asymptomatic patients; (iv) defaults in patient randomization and group or subgroup analysis due to limited or no data on the molecular analysis of tumors (with focus on biological characteristics related to tumor aggressiveness, invasiveness, and metastatic potential); and (iv) the remarkable variety regarding the methods of surveillance for cancer. To this end, it is necessary to conduct more well-designed studies.

**Figure 3:**
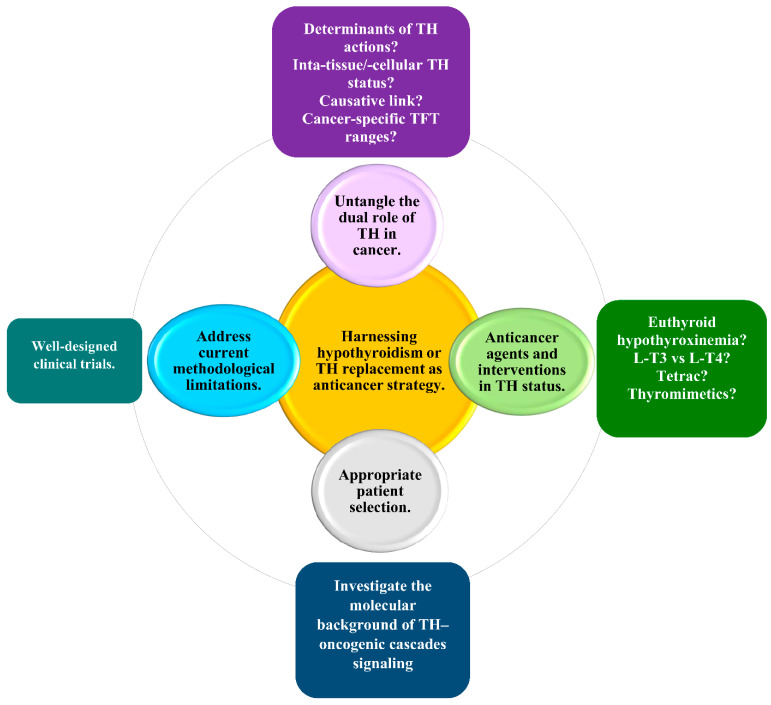
The four prerequisites for harnessing hypothyroidism, or THs replacement, as an anticancer strategy. The four circles represent the four prerequisites for harnessing hypothyroidism, or THs replacement, as an anticancer strategy, and the corresponding rectangles depict current challenges and future perspectives arising from each of these prerequisites. Abbreviations: THs, thyroid hormones; L-T3, liothyronine; L-T4, levothyroxine.

Figure 3 illustrates the four prerequisites for harnessing hypothyroidism, or THs replacement, as an anticancer strategy and the corresponding current challenges and future perspectives for a personalized approach.

Finally, to harness hypothyroidism therapeutically, it is essential to investigate the need for a specific TSH threshold in elderly patients. Generally, during the normal process of aging, normal serum TSH levels show an increasing trend even in the absence of underlying thyroid disease, comorbidities, or the administration of drugs interfering with the thyroid function. Whether reduced thyroid function in the elderly is merely a result of decreased metabolic demands or a protective mechanism against catabolic processes related to aging remains debatable. Although age-specific TSH reference ranges are still lacking, a target for serum TSH levels raised up to 4–6 mIU/L has been suggested by some experts for patients older than 65 years. Accumulating evidence indicates that the maintenance of serum TSH levels in the elderly, at a marginally higher level than younger individuals, may not subvert quality of life, cognitive function, and cardiovascular function, while also precluding the adverse events of overtreatment from the skeletal and the cardiovascular system. Until future, randomized controlled trials yield evidence-based guidelines for the management of hypothyroidism, with focus on mild subclinical hypothyroidism in older individuals with solid non-thyroid cancer, clinicians are encouraged to tailor THs replacement in this subpopulation according to the principal “first, do no harm”.

## 10. Conclusions

Standing at the crossroad of oncology and endocrinology, hypothyroidism in patients with solid non-thyroid cancer constitutes a unique entity. Their diagnosis and THs replacement are reminiscent of, but not identical to, their counterparts with respect to hypothyroidism in the general population. Due to contradictory clinical data, hypothyroidism cannot yet be established as a predictive and/or prognostic factor for solid non-thyroid cancer. To enable a personalized approach to the diagnosis and treatment of hypothyroidism, as well as to the exploitation of hypothyroidism, or THs replacement, as an anticancer strategy, many critical issues must be resolved, including: (i) the establishment of guidelines to be used as a starting point for tailored diagnosis and treatment; (ii) a clarification of the value of hypothyroidism as a predictive factor and/or as prognostic factor for solid non-thyroid cancer; (iii) the validation of patient-specific and cancer-specific factors that define whether THs exert tumor-promoting or anticancer effects; and (iv) the development and clinical evaluation of interventions in THs status and/or pharmaceutical agents to abrogate the tumor-promoting effects of THs and/or to mimic the anticancer effects of THs. The overarching aim is to ensure the maintenance of cancer patient quality of life in keeping with the notion of “treating patients not numbers” [51].

## Figures and Tables

**Table 1:** Anticancer treatment-related causes of hypothyroidism.

Anticancer Treatment	Incidence	Suggested Underlying Mechanisms	Screening Tests
Radiotherapy	20–60%	⮚ Primary hypothyroidism: Thyroid fibrosis, atrophy. Vascular proliferation. Autoimmunity. ⮚ Central hypothyroidism: Hypothalamus-pituitary involvement, especially in case of concurrent chemotherapy.	NTCP models are currently under evaluation.
Chemotherapy	NA due tofew cases	⮚ Cisplatin: In vitro cytotoxic effect on thyrocytes. In vitro inhibitory effect on cAMP, Tg, and T3 synthesis. ⮚ Mitotane: Inhibitory effect on both the secretory activity and the viability of pituitary TSH-secreting cells in mice. Central hypothyroidism of pituitary origin in ACC patients.	No specific recommendations.
IFN-α	2.4–31%	Induction of autoimmunity through the presentation of thyroid antigens to self-reactive lymphocytes by MHC class II molecules ectopically expressed on thyroid epithelial cells. High percentage of peripheral blood lymphocytes accompanied by ↑ NK lymphocytes and ↑ transitional B cells.	Baseline antithyroid antibody test.
HD IL-2	10–60%	Multifactorial background, including thyroid autoimmunity triggered by self-reactive lymphocytes and increased cytokines (e.g., IL-1, TNF-α, IFN-γ). Predisposing factor: preexistent thyroid antibodies.	Evaluation of TSH levels at baseline and Q 2–3 months during therapy.
Bexarotene	29–100%	⮚ Dose-dependent TSH suppression due to: TSHβ gene suppression. Altered pituitary setpoint for TSH secretion. Direct and rapid inhibition of TSH secretion. ⮚ ↑ THs degradation due to ↑ glucuronidation and sulfation, leading to ↓ THs levels.	⮚ L-T4 initiation simultaneously with bexarotene. Close monitoring of fT4 levels. Consider the need for higher than usual THs replacement dosages due to ↑ THs degradation.
ICPi	Anti-CTLA-4 mAbs: 0–11% Anti-PD-1/anti-PD-L1 mAbs: 2.5–10.19% Combination regimens: 4–27%	⮚ Ir primary hypothyroidism: De novo thyroid autoimmunity implicating antibody-mediated and T-cell- and NK-mediated mechanisms in predisposed patients ^a^. Silent inflammatory thyroiditis presented as thyrotoxicosis followed by hypothyroidism. ⮚ Ir secondary hypothyroidism ascribed to ir hypophysitis.	Evaluation of TSH and fT4 levels at baseline, at each course for 6 months, Q 2 courses for the next 6 months, and afterwards in case of clinical signs.
TKIs	Hypothyroidism: 2.3–92% Isolated TSH suppression: 1–33% ↓ TSH prior to ↑ TSH: 0–14%	Destructive thyroiditis. Capillary vasoconstriction in the thyroid. Decreased thyroid vasculature. Block of iodine uptake. Induction of positivity of anti-TPO Abs. Interaction with RAF kinases-mediated THs signaling pathways.	TSH evaluation at baseline and monitoring of patients treated with TKIs.
	• NA	Decreases iodide transport. Decreased iodide oxidation and organification (Wolff–Chaikoff effect). Decreased escape from Wolff–Chaikoff effect in patients with Hashimoto’s thyroiditis. Rapid inhibition of THs release. Immunostimulation. Decreased thyroid vascularization.	It is advisable to rule out thyroid pathology before contrast studies, especially in children, elderly patients, and patients with renal insufficiency.

^a^ Patient with autoimmune thyroiditis, or a history of hyperthyroidism, hemithyroidectomy, postpartum thyroiditis, subacute thyroiditis, or drug-induced thyroiditis. ↑, increased; ↓, decreased. Abbreviations: anti-CTLA-4 mAbs, anti-cytotoxic T-lymphocyte-associated antigen 4 monoclonal antibodies; anti-PD-1 mAbs, anti-programmed cell death 1 monoclonal antibodies; anti-PD-L1 mAbs, anti-PD-1 ligand 1 monoclonal antibodies; anti-TPO Abs, anti-thyroid peroxidase antibodies; ACC, adrenocortical cancer; f T4, free thyroxine; HD IL-2, interleukin 2; HLA-II, human leukocyte antigen class II; ICPi, immune checkpoint inhibitors; IL-1, interleukin 1; IFN-α, interferon type alpha; IFN-γ, interferon type gamma; Ir or ir, immune-related; L-T4, levothyroxine; NA, not applicable; NTCP, normal tissue complication probability; Q, every; THs, thyroid hormones; TKIs, tyrosine kinase inhibitors; TNF-α, tumor necrosis factor alpha; TSHβ, TSH beta-subunit.

**Table 2:** Clinical data indicating an association between hypothyroxinemia or hypothyroidism and favorable cancer prognosis.

Cancer Type	Study Type and Population	Results	Ref
Metastatic NSCLC	Case report.	⮚ Remission of metastatic NSCLC following recovery of amiodarone-induced myxedema coma.	[35]
Nonsurgical HCC	Retrospective study on 667 patients diagnosed at the Division of Gastroenterology and Hepatology/Medical University of Vienna between 1992 and 2012.	⮚ Univariate analysis: fT4 levels ≤ 1.66 vs. fT4 levels > 1.66 ng/dL: significantly improved median OS (10.6 months (95% CI, 7.5–13.6 months) vs. 3.3 months (95% CI, 2.2–4.3 months); *p* = 0.007).	[94]
NSCLC and SCLC (stages I–IV)	Retrospective case-control study assessing the occurrence of primary hypothyroidism among 1979 patients.	⮚ Stage I–IV disease: Hypothyroidism vs. euthyroidism: improved median survival (14.5 months vs. 11.1 months, respectively; *p* = 0.014). ⮚ Stage IV disease: Hypothyroidism vs. euthyroidism: improved median survival (11 months vs. 5 months; *p* = 0.0018).	[95]
High-grade optic glioma	Case report.	⮚ Hypothyroidism induced by antithyroid drugs in conjunction with carboplatin led to: Rapid response to carboplatin. Tumor regression within 4 weeks. Extended remission period (2.5 years). Prolonged OS (4.5 years).	[96]
Recurrent glioma treated with TMX	Phase I/II study on 22 patients in the US.	⮚ PTU-induced hypothyroidism compared to euthyroidism led to: Improved median survival: 10.1 months vs. 3.1 months; *p* = 0.03.	[97]
End-stage solid cancer (brain, ovary, lung, pancreas, salivary gland, breast cancer, mesothelioma, soft-tissue sarcoma)	Uncontrolled observational study on 23 patients.	⮚ Longer survival than 1-year estimated survival (20%) in patients with: MTM- and L-T3-induced euthyroid hypothyroxinemia. Replacement of L-T4 with L-T3 in case of preexisting primary hypothyroidism.	[98]
Metastatic triple negative breast cancer and pancreatic cancer	Case report.	⮚ Biochemical response of tumor to treatment with antithyroid drugs and exogenous administration of L-T3. ⮚ Inverse association of fT3 with tumor biomarkers. ⮚ Tumor progression and increase in tumor biomarkers alongside decreasing fT3 levels.	[99]
EC	A prospective study on 333 patients.Median FU: 35 months.	⮚ Hypothyroidism (TSH > 4.5 mU/L) compared to euthyroidism led to: ✓ Improved overall survival: HRAS: 0.34; 95% CI, 0.11–1.10; *p* = 0.07. Adjusted HR: 0.22; 95% CI, 0.06–0.74; *p* = 0.02. ✓ Improved cancer-specific survival: Adjusted HR: 0.21; 95% CI, 0.05–0.98, *p* = 0.04. ✓ Less recurrences: Adjusted HR: 0.17; 95% CI, 0.04–0.77; *p* = 0.02. Subclinical hypothyroidism compared to euthyroidism. ✓ Reduced risk of death: HR, 0.31; 95% CI, 0.09–1.28; *p* = 0.424. Adjusted HR: 0.54; 95% CI 0.12, 2.43; *p* = 0.503.	[100]
Solid cancers (lung cancer (69.9%), breast cancer (24.3%), melanoma (5.8%)) with newly diagnosed BMs	Evaluation of thyroid function in patients with BMs in a discovery cohort of 1692 patients and an independent validation cohort of 191 patients.	⮚ Discovery cohort: Significant association between hypothyroidism and favorable survival from diagnosis of cancer (31 vs. 21 months; *p* = 0.0026). BMs (12 vs. 7 months; *p* = 0.0079). Significant association of hypothyroidism with survival after diagnosis of BMs in multivariate analysis (HR, 0.76; 95% CI, 0.63–0.91; *p* = 0.0034). ⮚ Validation cohort: Significant association of hypothyroidism with improved survival from diagnosis of cancer (55 vs. 11 months; *p* = 0.00058). BMs (40 vs. 10 months; *p* = 0.0036).	[101]
Stage IV lung adenocarcinoma	Case report.	⮚ A hypothyroid 71-year-old man with stage IV lung adenocarcinoma who received no anticancer treatment and no THs replacement experienced survival longer than the median survival of lung adenocarcinoma (2.5 years versus 4–13 months, respectively). ⮚ The authors ascribed the prolongation of survival to hypothyroidism. ⮚ Repeated chest CT scans revealed decelerated, but not arrested, tumor growth.	[102]

Abbreviations: BC, breast cancer; BMs, brain metastases; CI, confidence interval; EC, endometrial cancer; ER, non-expressing estrogen receptor; f, free; FU, follow-up; HCC, hepatocellular cancer; HR, hazard ratio; L-T3, liothyronine; L-T4, levothyroxine; LNM, lymph node metastasis; MTM, methimazole; NSCLC, non-small-cell lung cancer; OS, overall survival; PR-, non-expressing progesterone receptor; PTU, propylthiouracil; Ref, reference; SCLC, small-cell lung cancer; T3, triiodothyronine; TMX, tamoxifen; vs., versus.

**Table 3:** Clinical data indicating hypothyroidism or decreased THs, even within the euthyroid range as predictors, of unfavorable prognoses.

Cancer Type	Study Type and Population	Results	Ref
Colorectal cancer	Case–control study of 273 cases.	⮚ SCH vs. euthyroidism: association with more advanced colonic lesions (*p* = 0.028) and colorectal cancer (*p* = 0.036).	[82]
Various cancertypes	⮚ Prospective study on 115,746 adult Taiwanese, of whom 1841 had SCH (TSH: 5.0–19.96 mIU/L) and 113,905 had normal thyroid function (TSH: 0.47–4.9 mIU). ⮚ FU: 1,034,082 person-years.	⮚ SCH vs euthyroidism: ▪ RRs of cancer-related deaths: All patients: 1.51 (95% CI, 1.06–2.15) Older patients: 1.71; 95% CI, 1.02–2.87) Females: 1.69; 95% CI, 1.08–2.65) Heavy smokers: 2.24; 95% CI, 1.19–4.21)	[90]
Nonsurgical HCC	⮚ Prospective study on 838 patients diagnosed with nonsurgical HCC. ⮚ Mean FU: 65.5 months.	⮚ Association between ↑ TSH levels and larger tumor size. ⮚ Significant association between ↑ TSH levels and worse outcomes in univariate analysis. ⮚ Median OS for TSH ≤ 1.7 mU/L vs. OS for TSH > 1.7 mU/L: 12.3 months (95% CI, 8.9–15.7 months) vs. 7.3 months (95% CI, 5.4–9.2 months) (*p* = 0.003). ⮚ Effect not confirmed in multivariate analysis after adjustment for other prognostic factors, including Child–Pugh class, tumor size, performance status, macrovascular invasion, extrahepatic spread, tumor treatment, AFP, and CRP levels.	[94]
Various cancer types	⮚ Prospective study on 212,456 middle-aged South Korean euthyroid men and women. ⮚ Median FU: 4.3 years.	⮚ Association between fT4 values in 2nd fT4 tertile vs. fT4 levels in 1st fT4 tertile and ↓ breast cancer mortality (HR, 0.49; 95% CI, 0.28–0.84). ⮚ Inverse association between fT3 levels and cancer mortality (HR, 0.62; 95% CI, 0.45–0.85; p for linear trend = 0.001. ⮚ Inverse association between fT3 and fT4 and liver cancer mortality (HR per SD change: 0.64 for fT3, 0.52 for fT4).	[103]
EC	⮚ Retrospective analysis of 199 patients included in an Austrian multicenter trial.	⮚ Association between ↑ TSH and unfavorable disease-specific survival in univariate (*p* = 0.01) and multivariate (*p* = 0.03) survival analyses.	[104]
HCC	⮚ Study on a retrospective cohort (*n* = 1005) at Beijing Ditan Hospital and a prospective cohort (*n* = 77) to develop and validate a novel prognostic model.	⮚ Superiority of a model comprising TSH levels and three more variables over conventional scoring systems in predicting PFS for hepatocellular cancer ⮚ KM curve: ↑ TSH level group vs. ↓ TSH level group shorter PFS (*p* = 0.001).	[105]
Various cancer types.	⮚ Systematic analysis of nine studies: two case–control studies, three retrospective cohort studies, and four prospective cohort studies.	⮚ SCH vs. euthyroidism: ↑ cancer-related mortality specifically for colorectal cancer.	[106]

↑, increased; ↓, decreased. Abbreviations: AFP, α-fetoprotein; CRP, C-reactive protein; EC, endometrial cancer; CRC, colorectal cancer; f, free; fT3, free triiodothyronine; fT4, free thyroxine; FU, follow-up; HCC, hepatocellular cancer; HR, hazard ratio; KM, Kaplan–Meier; OS, overall survival; PFS, progression-free survival; Ref, reference; RR, relative risk; SCH, subclinical hypothyroidism; SD, standard deviation; T3, triiodothyronine; T4, thyroxine; TSH, thyroid-stimulating hormone; vs., versus.

**Table 4:** Data indicative of no significant association between hypothyroidism or increasing TSH and cancer outcome.

Cancer Type	Study Type and Population	Results	Ref
Breast cancer	⮚ Prospective cohort study on 134,122 multiethnic postmenopausal women in the US, aged 50 to 79 years. ⮚ Recruitment of participants from 40 clinical centers in the US between 1 October 1993 and 31 December 1998. ⮚ Initial FU through March 2005. ⮚ Two extension studies (2005–2010 and 2010–2020).	⮚ No significant impact of THs status on SEER stages, histologic types, morphologic grades, ER status, PR status, or HER2 status of breast cancer	[75]
Various cancer types	⮚ Prospective study on 212,456 middle-aged South Korean euthyroid people. ⮚ Median FU: 4.3 years.	⮚ TSH levels showed no association with mortality endpoints.	[103]
Various cancer types	⮚ Systematic analysis of nine studies: two case–control studies, three retrospective cohort studies, and four prospective cohort studies.	⮚ No association between SCH and cancer mortality among men aged ≥65 years.	[106,107]
Various cancer types	⮚ Prospective study on 1587 participants from the Osteoporotic Fractures in Men (MrOS) study—a cohort of community-dwelling US men aged 65 years and older. ⮚ Mean FU: 8.3 years.	⮚ Fully adjusted models: no association between baseline TSH levels and cancer death (HR, 0.96 per mIU/L; 95% CI, 0.85–1.07).	[107]
Various cancer types	⮚ Prospective study on 75,076 women aged 20–89 years certified as radiologic technologists in the US in 1926–1982, who completed baseline questionnaires in 1983–1998, with no malignant disease or benign thyroid disease apart from thyroid dysfunction. ⮚ FU ≥ 10 years (through the Social Security Administration database and the National Death Index-Plus).	⮚ No increased cancer-related mortality in hypothyroid women	[108]
Breast cancer	⮚ Prospective study on 2185 people included in the Swedish Malmö Preventive Project, in whom T3 levels were measured before a diagnosis of breast cancer. ⮚ Mean FU: 23.3 years.	⮚ No statistically significant association between T3 levels and deaths due to cancer types other than breast cancer (age-adjusted HR,1.09; 95% CI, 0.72–1.65) or all-cause mortality (HR, 1.25; 95% CI, 0.97–1.60).	[109]
Breast cancer	⮚ A retrospective cohort study on 437 patients of whom 422 (97%) had complete AJCC staging data and 420 (96%) had complete tumor grade data from 2002 to 2014. Of these patients, 95.7% were euthyroid based on the weighted TSH (0.3–4.7).	⮚ TSH concentration as a continuous variable. ⮚ No association between increasing serum TSH levels within normal reference ranges and tumor grade, AJCC stage, tumor size, or individual staging elements (T, N, M). ⮚ TSH concentration as a categorical variable: no significant correlation between serum TSH concentration and any markers of breast cancer aggressiveness.	[110]
Breast cancer	⮚ A prospective study on 35,463 Danish women, aged 35 years or older, diagnosed with stage I–III operable breast cancer between 1996 and 2009. ⮚ FU: 212,641 person-years	⮚ Prevalent hypothyroidism: 1272 women. ⮚ Incident hypothyroidism: 859 women. ⮚ Recurrent breast cancer: 5810 patients. ⮚ No association between either prevalent or incident hypothyroidism and recurrence (adjusted HR prevalent, 1.01, 95% CI 0.87–1.19; adjusted HR incident, 0.93, 95% CI, 0.75–1.16). ⮚ Confirmation of results after stratification by menopausal status, ER status, chemotherapy, or radiotherapy.	[111]

Abbreviations: AJCC, American Joint Committee on Cancer; CI, confidence interval; ER, estrogen receptor; FU, follow-up; HER2, human epidermal growth factor receptor 2; HR, hazard ratio; PR, progesterone receptor; Ref, reference; RH, relative hazard; SEER, Surveillance, Epidemiology, and End Results; THs, thyroid hormones; TSH, thyroid-stimulating hormone.
